# PD-L2 Blockade Exacerbates Liver Lesion in Mice Infected with *Capillaria hepatica* through Reducing Alternatively Activated Macrophages

**DOI:** 10.3390/tropicalmed8010046

**Published:** 2023-01-06

**Authors:** Minjun Huang, Xiaoli Li, Xiaoyan Zheng, Fei Wang, Yang Zou, Lei Wang

**Affiliations:** 1 Beijing Institute of Tropical Medicine, Beijing Friendship Hospital, Capital Medical University, Beijing 100050, China; 2 Beijing Key Laboratory for Research on Prevention and Treatment of Tropical Diseases, Beijing 100050, China

**Keywords:** alternatively activated macrophages, *Capillaria hepatica*, immunomodulation, programmed death ligand 2

## Abstract

*Capillaria hepatica* is a seriously neglected zoonotic parasite, which infects the liver of mammalian hosts, causing fibrosis or even hepatic failure. At present, the immune responses elicited by *C. hepatica* are not fully understood, and the role(s) of the programmed death 1 (PD-1) signaling pathway in the context of *C. hepatica*-induced pathology are not known. In this study, we identify that the late stage of infection with *C. hepatica*—especially the egg-derived antigens—modulates the host immune responses to promote alternatively activated macrophage (M2) polarization and programmed death ligand 2 (PD-L2) expression. The PD-L2-expressing alternatively activated M2 macrophages play an important role in maintaining Th2-biased regulatory immune responses, which may facilitate the survival of parasitic worms or eggs within the infected liver and reduce the liver pathology caused by the egg granulomas. Treatment with anti-PD-L2 antibody had no effect on the survival of parasitic eggs but deteriorated the pathology of egg granulomas. The obtained results suggest that PD-1/PD-L2 signaling, which is involved in alternative macrophage polarization, determines the immune response pattern and the immunopathology, consequently determining the outcome of the parasitic infection.

## 1. Introduction

*Capillaria hepatica* is a zoonotic nematode which causes hepatic capillariasis in rodents and other mammal species, including humans. The nematode infection results in a serious hepatic disorder with liver functional damage, granulomatous inflammation, fibrosis, or even death [1]. Due to the rarity of infection in humans and the associated non-specific clinical manifestations, *C. hepatica* infection has been seriously neglected and is often misdiagnosed as other diseases.

Humans acquire *C. hepatica* infection through ingestion of embryonated eggs, due to the contamination of water or food. The larvae hatch and invade the intestinal mucosa, and then enter the liver through the mesenteric vein and portal vein system. Adult worms parasitize in the liver of the mammalian host, where the females lay eggs in the liver parenchyma after mating. The unembryonated eggs are passed into the environment, either through the death and decomposition of the infected animal, or the feces of its predator/scavenger, to develop into embryonated eggs [2].

Egg granulomas and liver fibrosis have been observed in the hepatic pathology of humans infected with *C. hepatica* in our previous study [3]. The infiltration of inflammatory cells around eggs forms granulomas and fibrosis, which eventually lead to an irreversible impairment of the liver. Except for the tissue damage caused by the helminth infection in the parasitized host, the helminths themselves have also developed an immune evasion mechanism to reduce host immune attack as a survival strategy. This defense mechanism includes the secretion of functional molecules that play an immunomodulatory role in the host immune response [4]. As a result, the reduced immune response to the helminth infection may benefit the hosts themselves, with reduced immunopathology and less tissue damage caused by the infection [5]. The helminth-induced immunomodulation may benefit the host by restricting the helminth burden and preventing continual infection, which is called concomitant immunity [6]. Several studies have demonstrated that hepatotropic parasites, including *Schistosoma japonicum*, *Schistosoma. mansoni*, or *Fasciola hepatica,* activate the immunomodulatory pathway to facilitate their parasitism in the host [7,8,9]. Recently, PD-1 and its ligands 1 and 2 (PD-L1/L2) have been identified as part of the immunomodulatory pathways involved in peripheral tolerance and immune escape mechanisms during chronic viral infections and cancer development [10]. The PD-1 pathway has been shown to be significantly up-regulated in macrophages and dendritic cells during protozoa and helminth infections—such as those associated with *Plasmodium* [11], *Leishmania* spp. [12], *Schistosoma japonicum* [13], *Fasciola. hepatica* [14], and *Trichinella spiralis* [15]—suggesting its involvement in immune modulation of the host immune response. In addition, helminth infections stimulate alternatively activated macrophages (M2) in infected tissues. In particular, helminth-derived proteins stimulated PD-1 expression on M2 macrophages, thus reducing inflammatory bowel disease, indicating that the PD-1 pathway is involved in the helminth-induced M2 polarization during helminth infections [15]. Some studies have shown that a *C. hepatica* infection induced an immune response in the early stage [16,17]; however, little is known regarding its effect on the host immune response during chronic infection, and whether the PD-1 signaling pathway is involved in the pathological change caused by the infection.

In this study, we identify that chronic infection of *C. hepatica* induces alternatively activated macrophages (M2) by activating the PD-1/PD-L2 pathway. In particular, a PD-L2 blockade aggravated liver lesions caused by the egg granulomas and promoted liver fibrosis associated with the shift of M2 to M1 in the liver infected with *C. hepatica*.

## 2. Materials and Methods

### 2.1. Parasite

*C. hepatica*-infected mice were donated by Dr. Yalan Zhang (Henan Institute of Parasitology, Henan Center for Diseases Prevention and Control, Zhengzhou, China). To obtain embryonated eggs, a previously described method was applied with minor modifications [18]. Briefly, the liver collected from a *C. hepatica*-infected mouse was homogenized; the unembryonated eggs were collected by filtering the liver homogenate through a 500 mm filter and washed three times with centrifugation at 800 × *g* for 2 min. The unembryonated eggs in the pellet were suspended in PBS-containing penicillin (100 U/mL) and streptomycin (100 µg/mL) and incubated at 30 °C for 28–30 days. The embryonated eggs were identified and counted under a microscope.

### 2.2. Mice and C. hepatica Infection

Female six- to eight-week-old BALB/c mice were purchased from the Laboratory Animal Services Center of the Capital Medical University (Beijing, China) and were maintained under specific pathogen-free conditions at suitable humidity and temperature.

A total of 50 BALB/c mice each received 200 embryonated eggs of *C. hepatica* in 0.5 mL of PBS buffer by oral gavage. The same number of control mice received 0.5 mL of PBS only. Five mice from each group were sacrificed at 0, 3, 6, 9, and 12 weeks post-infection. The sera, livers, and spleens were harvested for further analysis.

### 2.3. Preparation of Adult Worm Extracts (AWE) and Egg Extracts (EE) from C. hepatica

The adult worms and eggs were isolated from the liver parenchyma of mice infected with *C. hepatica* for 2 weeks and 8 weeks, respectively, after being euthanized with an overdose of ethyl ether. The worms or eggs were washed three times in PBS, then homogenized by ultrasonication under ice. The extract supernatants were collected by centrifuging at 18,000× *g* for 10 min, followed by filtering through a 0.45 μm filter to obtain the AWE and EE. The concentration of protein was measured using the Bradford assay reagent (Thermo Fisher Scientific, Waltham, MA, USA).

### 2.4. Liver Macrophage Isolation

The macrophages were isolated from infected or normal mice as previously described with modification [19]. Briefly, liver tissues were collected from the euthanized mice and minced into small pieces through 100 µm nylon meshes. The red blood cells were removed using a lysis buffer (Sigma Aldrich, St. Louis, MO, USA). The liver homogenate was incubated in RPMI 1640 containing 0.1% type IV collagenase at 37 °C for 1 h. The liver single-cell suspension was acquired through centrifugation at 500× *g* for 10 min at 4 °C, then re-suspended in DMEM with 10% fetal bovine serum, 100 U/mL penicillin, and 100 µg/mL streptomycin, and cultured in a T25 culture flask. The non-adherent cells were removed from the culture flask after being incubated for 2–4 h at 37 °C. The adherent cells were collected as hepatic macrophages, confirmed by flow cytometry labeled with FITC-F4/80.

### 2.5. Hepatic Macrophages Cultured In Vitro and Cytokine Measurement

A total of 1 × 10^6^ hepatic macrophages collected from the livers of the normal or infected mice were cultured in an RPMI 1640 medium containing 10 μg/mL of AWE or EE for 72 h at 37 °C, 5% CO_2_. The same number of hepatic macrophages were incubated with 100 ng/mL LPS (Sigma Aldrich, MO, USA) or 10 ng/mL IL-4 (Peprotech Inc., Rocky Hill, NJ, USA), as a control, for M1 and M2 macrophage activation, respectively. The supernatants from the culture were collected and the cytokines (including IFN-γ, TNF-α, IL-10, and IL-13) were determined by an enzyme-linked immunosorbent assay (ELISA), according to the manufacturer’s instructions (Dakewe, Shenzhen, China).

### 2.6. Flow Cytometry

To examine PD-L1, PD-L2, and PD-1 expression, the hepatic macrophages or splenocytes were collected from the normal or *C. hepatica*-infected mice and adjusted to 1 × 10^6^ cells/100 μL in PBS containing 1% FBS. Single-cell suspensions were then blocked with anti-CD16/32 mouse Fc Block (BioLegend, San Diego, CA, USA) and analyzed for the expression of cell surface markers using combinations of the following antibodies: FITC-conjugated anti-F4/80; APC-conjugated anti-CD86 or anti-CD206; PE-conjugated anti-PD-L1; PerCP/Cyanine 5.5-conjugated anti-PD-L2; FITC-conjugated anti-CD3; APC-conjugated anti-CD8 or anti-CD4; or PE-conjugated anti-PD-1 (R&D system, Minneapolis, MN, USA). Rat IgG2a isotype controls were added and run with each set of samples to define negative and positive cell populations.

To evaluate the intracellular expression of IFN-γ in the splenocytes from the mice, spleen cells were initially stimulated with 25 ng/mL of phorbol myristate acetate (Sigma-Aldrich, St. Louis, MO, USA) and 1 μg/mL of brefeldin A (BD Pharmingen, San Diego, CA, USA) for 4 h at 37 °C, 5% CO_2_, then strained with FITC-conjugated anti-CD3. Once fixed and permeabilized, PE-conjugated anti-IFN-γ or rat IgG2a isotype control antibody was added. The stained cell suspensions were analyzed using a BD FACSCalibur Flow Cytometer (BD Biosciences, San Jose, CA, USA). The results were analyzed using the FlowJo software (BD Biosciences, San Jose, CA, USA).

### 2.7. Quantitative Real-Time PCR (RT-qPCR) Analysis

The total RNA was isolated from the hepatic macrophages using TRIzol reagent (Invitrogen, Carlsbad, CA, USA), and the total cDNA was reverse-transcribed from the total RNA using a reverse transcription kit, according to the manufacturer’s instructions (Fermantas Life Sciences, Burlington, Ontario, CA). An RT-qPCR was conducted using SYBR Green Master reagents (Roche Diagnostics, Basel, Switzerland) in a 7300 Real-Time PCR machine (Applied Biosystems, Foster City, CA, USA). The fold changes in gene expression, compared to the housekeeping gene (β-actin), were calculated using the 2^−ΔΔCt^ method.

The sequences of the primer pairs used in RT-qPCR are as follows:

iNOS: forward 5′-CTGGAGGAGCTCCTGCCTCATG-3′, reverse 5′-GCAGCATCCCCTCTGATGGTG-3′

Arginse-1: forward 5′-GTATGACGTGAGAGACCACG-3′, reverse 5′-CTCGCAAGCCAATGTACACG-3′

IFN-γ: forward 5′-GCTGCTGATGGGAGGAGATG-3′ reverse 5′-TGTCTGGCCTGCTGTTAAAGC-3′

TNF-α: forward 5′-TTCTGTCTACTGAACTTCGGGGTGATCGGTCC-3′, reverse 5′-GTATGAGATAGCAAATCGGCTGACGGTGTGGG-3′

IL-10: forward 5′-TCTATTCTAAGGCTGGCCACACT-3′, reverse 5′-CAATTGAAAGGACACCATAGCAAA-3′

IL-13 forward 5′-AGACCAGACTCCCCTGTGCA-3′, reverse 5′-TGGGTCCTGTAGATGGCATTG-3′

β-actin: forward 5′-ATGGATGACGATATCGCT-3′, reverse 5′-ATGAGGTAGTCTGTCAGGT-3′.

### 2.8. PD-L2 Blocking Management In Vivo

Five mice from each group received intraperitoneal injections of 500 μg anti-mouse PD-L2 monoclonal antibody (mAb; MIH18, Mouse IgG1; Biolegend) on day 5 after *C. hepatica* infection, followed by an injection of 250 μg on day 8. The control mice were given the same amount of isotype IgG (MG1-45; Biolegend) in the same regime.

### 2.9. Histochemical Staining of Liver Tissue

The livers were collected from the mice infected with *C. hepatica* for 8 weeks and fixed in 4% buffered formalin. The liver tissue sections were stained with hematoxylin and eosin (H&E). The density of the hepatic granulomas in the liver was evaluated by stereological point-counting [20]. Basically, it was estimated from the number of granulomas observed in the grid point intersections divided by the number of grid points intersecting all the liver sections. The grid points were generated using ImageJ analysis software. The immunohistological, semi-quantitative analysis for hepatic fibrosis was evaluated by staining with Masson’s trichrome (Sigma-Aldrich, St. Louis, MO, USA) and sirius red Sigma-Aldrich, St. Louis, MO, USA). The area percentage of positive staining for the Masson trichrome and sirius red in five different fields for each section was determined to quantify the collagen deposition using the ImageJ software. The proportion of positively stained areas was calculated as follows: (collagen area/total area-vascular lumen area) × 100%.

### 2.10. Assessment of Egg Burden

One gram of liver collected from each infected mouse was digested with 5% KOH at 37 °C overnight, following which the number of eggs per gram of liver was determined through microscopic examination [13].

### 2.11. Statistical Analyses

Statistical analyses were performed with one-way ANOVA using the SPSS version 24.0 software (SPSS, Chicago, IL, USA). All the data are expressed as the mean ± standard error of the mean (SEM), with differences considered significant when *p* was less than 0.05.

## 3. Results

### 3.1. C. hepatica Infection Induced Macrophages Polarization

**Figure 1:**
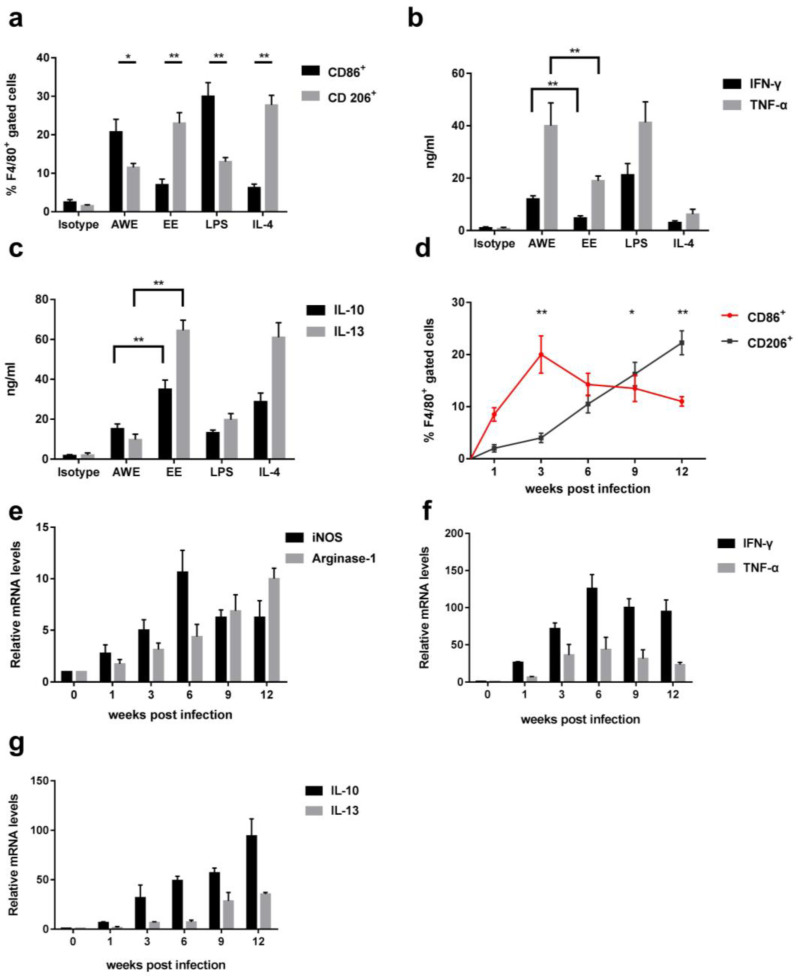
*C. hepatica* infection induced macrophage polarization in livers of infected mice: (**a**) Macrophages collected from livers of normal mice were incubated in vitro with *C. hepatica* AWE or EE, which induced M1 (F4/80^+^CD86^+^) and M2 (F4/80^+^CD206^+^) phenotypes, as measured by flow cytometry, respectively. Isotype antibody was used as negative control; LPS or IL-4 co-cultured with macrophages as M1 and M2 positive controls, respectively. (**b**) The levels of M1-related cytokines IFN-γ and TNF-α were measured in the supernatant of normal mouse hepatic macrophages incubated with AWE or EE by ELISA. (**c**) The M2-related cytokines IL-10 and IL-13 were measured in the supernatant of normal hepatic macrophages incubated with AWE and EE of *C. hepatica* by ELISA. (**d**) Macrophage polarization from M1 (CD86^+^) to M2 (CD206^+^) on gated F4/80^+^ intrahepatic macrophage cells during the natural course of *C. hepatica* infection in mice. (**e**) iNOS (M1) and Arginase-1 (M2) dynamic mRNA expression in macrophages collected from livers of mice infected with *C. hepatica* measured by RT-qPCR relative to the expression of β-actin (control gene). (**f**) M1-related IFN-γ and TNF-α dynamic mRNA expression in the intrahepatic macrophages from *C. hepatica*-infected mice. (**g**) M2-related IL-10 and IL-13 dynamic mRNA expression in the intrahepatic macrophages from *C. hepatica*-infected mice. Data are expressed as mean ± SEM from three independent experiments (*n* = 5 mice per group). * *p* < 0.05, ** *p* < 0.01.

The macrophages collected from the mouse liver tissue were incubated with either AWE or EE (each 10 µg/mL) of *C. hepatica* for 72 h in vitro. The flow cytometric analysis revealed that the incubation with AWE significantly increased the expression of F4/80^+^CD86^+^ (M1) on the surface of macrophages, while the EE mostly stimulated the expression of F4/80^+^CD206^+^ (M2). There was no significant increase of CD86^+^ or CD206^+^ expression on the surface of macrophages incubated with an isotype control. As expected, LPS stimulated the expression of F4/80^+^CD86^+^ and IL-4 stimulated F4/80^+^CD206^+^, as positive controls (Figure 1a). The results indicated that the AWE mostly stimulated M1 macrophage differentiation, while the EE stimulated M2 macrophages. The cytokine profile in the supernatants of the treated macrophages was correlated with the expression of CD86^+^ and CD206^+^. The macrophages treated with AWE secreted a higher level of M1 cytokines (IFN-γ, TNF-α) than those treated with EE, while the EE stimulated a higher level of M2 cytokines (IL-10, IL-13), compared to the AWE (Figure 1b,c). These data suggest that adult worm antigens mainly induced M1 macrophages and EE induced the M2 macrophage phenotypes.

The effect of *C. hepatica* antigen stimulation on macrophage polarization was also confirmed in mice infected with *C. hepatica* in vivo. The macrophages extracted from the livers of mice infected with *C. hepatica* expressed high levels of F4/80^+^CD86^+^, where the expression reached its peak at 3 weeks post-infection, coincident with the development of adult worms in the liver parenchyma. After that, the level of F4/80^+^CD86^+^ expression declined over time. However, the expression of F4/80^+^CD206^+^ started to increase 3 weeks post-infection and was maintained until the end of the experiment (12th week), as correlated with the time period of the deposition of eggs laid by the gravid female worms in the liver tissue (Figure 1d). In addition, the transcriptional levels of the M1 marker, iNOS, and the M2 marker, Arginase-1, in the macrophages collected from the infected livers, as measured by RT-qPCR, showed the same trends as the CD86^+^ and CD206^+^ expression during the infection course. The transcription of the iNOS gene increased upon infection and reached its highest level at the 6th week post-infection, then decreased; while the Arginase-1 started to increase in the 3rd week and reached the highest level at the endpoint of the 12th week post-infection (Figure 1e), further suggesting that adult *C. hepatica* worms stimulated the M1 macrophages, while the eggs boosted the M2 macrophages in the infected liver tissue. The dynamic changes from M1 to M2 upon the occurrence of eggs being laid by the gravid female worms in the liver were also supported by the cytokine profiles measured in the liver-isolated macrophages by RT-qPCR. The M1-related cytokines, IFN-γ and TNF-α, increased and reached a peak at the 6th week post-infection (with IFN-γ higher than TNF-α), while the M2-related cytokines, IL-10 and IL-13, were boosted during the late stage of infection (with higher IL-10 than IL-13; Figure 1f,g). All the data from both in vitro and in vivo studies supported that adult worms or worm antigens mainly stimulated M1 macrophages, while eggs or egg-related antigens stimulated M2 macrophage polarization in the infected liver during the natural infection course of *C. hepatica*.

### 3.2. Chronic C. hepatica Infection-Induced M2 Macrophage Polarization through PD-1/PD-L2 Pathway

**Figure 2:**
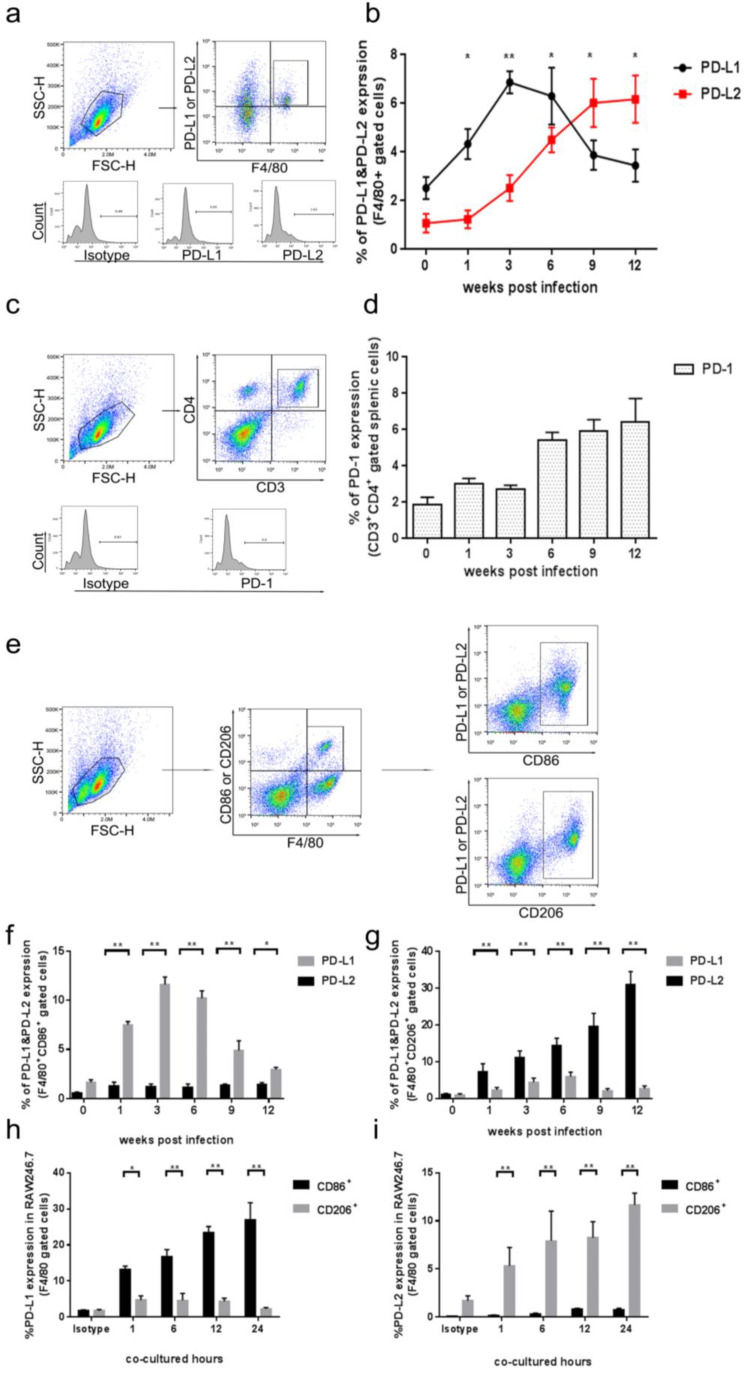
Late stage of *C. hepatica* infection or egg-derived antigens induced M2 macrophages through PD-1/PD-L2 pathway. Flow cytometry was performed to measure the expression of PD-L1 and PD-L2 on F4/80^+^CD86^+^ (M1) or F4/80^+^CD206^+^ (M2) macrophages: (**a**) Flow cytometry gating strategy to define F4/80^+^ macrophage from the infected liver tissues. (**b**) PD-L1 and PD-L2 expression on the macrophages (F4/80^+^) collected from livers of mice infected with *C. hepatica*. (**c**) Flow cytometry gating strategy to define CD3^+^ CD4^+^ infected splenic T-cells. (**d**) PD-1 expression on the CD3^+^CD4^+^ T-cells of spleens from mice infected with *C. hepatica*. (**e**) Flow cytometry gating strategy to define F4/80^+^CD86^+^ or F4/80^+^CD206^+^ cells from infected liver tissues or RAW246.7 cell line. (**f**) PD-L1 and PD-L2 expression on the macrophages expressing F4/80^+^CD86^+^ (M1) collected from livers of mice infected with *C. hepatica*. (**g**) PD-L1 and PD-L2 expression on the macrophages expressing F4/80^+^CD206^+^ (M2) collected from livers of mice infected with *C. hepatica*. (**h**) PD-L1 expression on the M1 (F4/80^+^CD86^+^) and M2 (F4/80^+^CD206^+^) RAW246.7 cell line co-incubated with AWE in vitro. (**i**) PD-L2 expression on the M1 (F4/80^+^CD86^+^) and M2 (F4/80^+^CD206^+^) RAW246.7 cell line co-incubated with EE in vitro. Data are expressed as mean ± SEM from three independent experiments (*n* = 5 mice per group). * *p* < 0.05, ** *p* < 0.01.

To determine whether the PD-1/PD-L pathway is involved in macrophage polarization in the liver during *C. hepatica* infection in mice, the expression levels of PD-L1 and PD-L2 on the surface of macrophages (F4/80^+^ gated) isolated from the livers of mice infected with *C. hepatica* at different time points were evaluated kinetically by flow cytometry. The results demonstrated that the PD-L1 expression was initially stimulated upon infection and reached a peak in the third week—which was correlated with the development of adult worms in the liver—and then declined during the late infection period (Figure 2a,b). On the contrary, the PD-L2 expression on the macrophages was initiated upon the development of adult worms and eggs being laid in the livers of the infected mice (3 weeks post-infection), and reached the highest level at the end of the infection (9–10 weeks post-infection; Figure 2a,b). These results suggest that *C. hepatica* adult worms mainly stimulated PD-L1 on the macrophages, while the eggs or egg-derived products mainly stimulated PD-L2 expression on the macrophages during *C. hepatica* infection in the liver. The PD-L2 expression on the macrophages as a result of the eggs laid by the adult worms in the liver was correlated with the PD-1 expression on splenic CD3^+^CD4^+^ T-cells, which increased at 3 weeks post-infection and reached a peak at the end of infection at the 12th week (Figure 2c,d). These results clearly suggest that the PD-1/PD-L2 axis is involved in the immune regulation at the late stage of *C. hepatica* infection in the liver when the eggs start to be deposited in the hepatic parenchyma. Even though the PD-L1 expression was stimulated on the macrophages during the early infection, PD-1 was not significantly stimulated on the CD3^+^CD4^+^ T-cells, accordingly, at the same time.

To further determine the specific M1 or M2 macrophages related to the expression of PD-L1 or PD-L2 during the infection of *C. hepatica*, the macrophages were collected from the livers of the infected mice and the expressions of PD-L1 and PD-L2 were measured on the macrophages gated with F4/80^+^CD86^+^ (M1) or F4/80^+^ CD206^+^ (M2). The flow cytometry revealed that PD-L1 was stimulated on the M1 macrophages (CD86^+^) at the early stage of infection and reached a peak at the 3rd week of infection. However, the expression of PD-L2 was stimulated upon infection with *C. hepatica* and increased gradually to the highest point at the termination of the infection (12th week post-infection; Figure 2e–g), further suggesting that M2 macrophages and the PD-1/PL-2 pathway are mainly involved in the regulation and modulation of immune responses to the infection of *C. hepatica*, especially to the eggs of the worm in the late stage of infection.

The *Capillaria hepatica* adult worms induced PD-L1 expression on M1 macrophages, while the eggs induced PD-L2 expression on M2 macrophages. This was further confirmed by the incubation of macrophages (RAW246.7 cell line) with *C. hepatica* AWE or EE in vitro. Flow cytometry revealed that PD-L1 was up-regulated only on CD86^+^ cells by AWE antigens, while PD-L2 was up-regulated only on CD206^+^ cells by EE antigens up to 24 h after incubation, further confirming that egg-derived products induce M2 polarization through the PD-1/PD-L2 pathway (Figure 2e,h,i). There was no effect of AWE on the expression of PD-L2 or EE on PD-L1 on different phenotype macrophages at different culture time points (Supplementary Figure S1).

Taken together, these results suggest that *C. hepatica* infection regulates the host immune response through egg-derived antigens by the PD-1/PD-L2 pathway on the M2 macrophages.

### 3.3. PD-L2 Blockade Exacerbated Egg Granuloma and Fibrosis in Infected Liver

**Figure 3:**
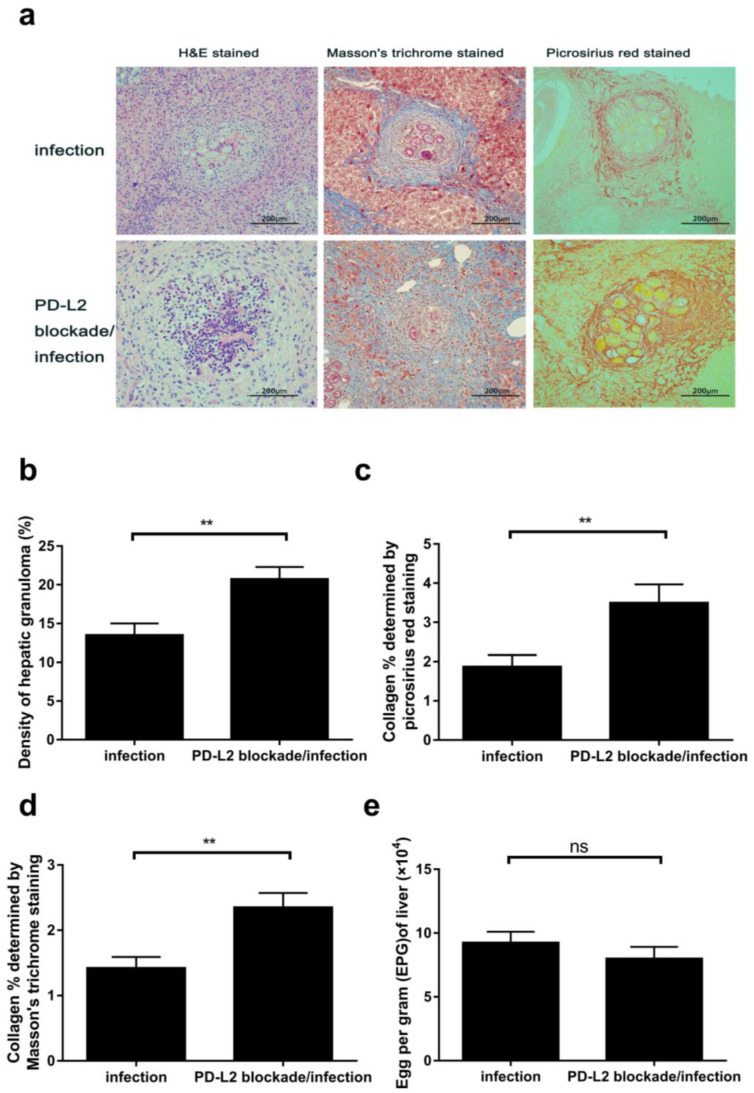
PD-L2 blockade deteriorated hepatic pathology of mice infected with *C. hepatica:* (**a**) Representative histopathological sections of livers of mice infected with *C. hepatica* treated with anti-mouse PD-L2 monoclonal antibody or isotype control. Liver sections were stained with H&E, Masson’s trichrome, or Sirius Red to reveal the inflammatory cell infiltration and collagen deposition within the egg granulomas. Images are representative of three independent experiments and observed by microscopy at 200× magnification. (**b**) The density of hepatic granulomas measured by stereological point-counting (SPC) in the sections of mice treated with anti-PD-L2 or not [20]. (**c**) The average area of collagen stained with Sirius Red in livers of experimental mice from four view fields, performed by the ImageJ software (version 1.48). (**d**) The average area of collagen stained with Masson’s trichrome stain in liver tissue from four view fields, performed by the ImageJ software. (**e**) Eggs per gram (EPG) in livers of mice treated with anti-PD-L2 antibody or isotype control. Data are expressed as mean ± SEM from three independent experiments (*n* = 5 mice per group). ** *p* < 0.01 between two groups. ns, not significant.

To determine whether PD-L2 is involved in the regulation of immunopathology in the livers of mice infected with *C. hepatica*, the infected mice were each treated with 500 μg anti-mouse PD-L2 monoclonal antibody intraperitoneally on day 5 after the *C. hepatica* infection, followed by 250 μg on day 8. The mice were euthanized, and their livers were collected. A histopathological examination of the livers from the mice infected with *C. hepatica* demonstrated that many egg granulomas had developed in the liver tissue, with many inflammatory cells infiltrated around the eggs (stained with H&E), and some collagen deposited around the granuloma (stained with Masson’s trichrome and Picrosirius red; Figure 3a). Interestingly, treatment with anti-PD-L2 monoclonal antibody significantly increased the infiltration of the inflammatory cells around the egg granuloma, and more collagen was deposited within the granulomas, compared to those receiving the isotype control. The increased inflammatory cells were observed in each granuloma. The collagen deposition area was also significantly increased in mice treated with anti-PD-L2 antibody, compared to those without treatment (Figure 3b–d). However, there was no reduction in the number of *C. hepatica* eggs observed in the livers of infected mice receiving PD-L2 monoclonal antibody, compared to those receiving PBS only (Figure 3e). The results clearly indicate that blocking the PD-L2 pathway significantly exacerbated the inflammation and fibrosis of egg granulomas developing in the liver of mice infected with *C. hepatica* but had no effect on reducing the egg count.

### 3.4. PD-L2 Blockade Increased Inflammatory Immune Responses to C. hepatica Infection In Vivo

**Figure 4:**
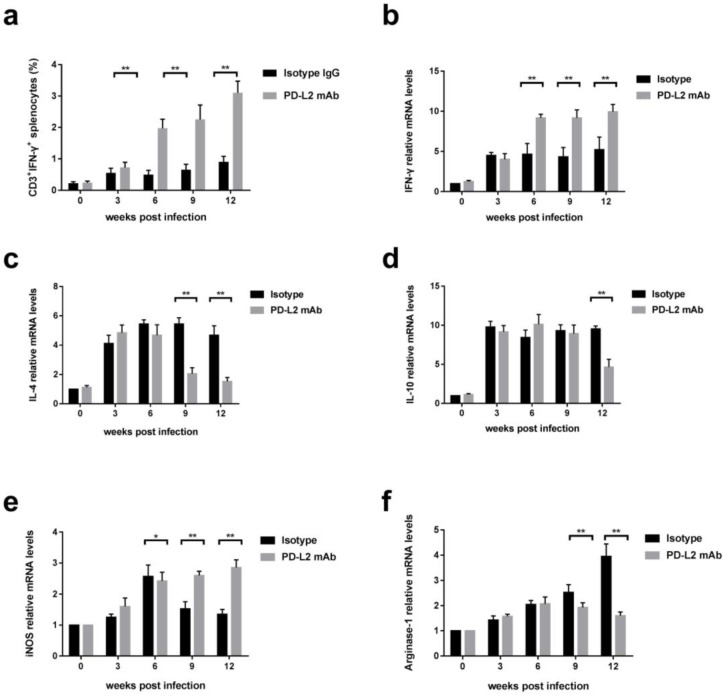
PD-L2 blockade increased inflammatory immune responses to *C. hepatica* infection in vivo. After being treated with PD-L2 monoclonal antibody, the percentage of CD3^+^IFN-γ^+^ T cells (**a**) was measured in splenocytes of mice infected with *C. hepatica* by flow cytometry. The mRNA expression levels of IFN-γ (**b**), IL-4 (**c**), and IL-10 (**d**) were measured in liver tissue by RT-qPCR, relative to the housekeeping gene β-actin. The transcriptional levels of iNOS (**e**) and Arginase-1 (**f**) were also measured in macrophages collected from liver tissue of mice upon treatment of PD-L2 antibody by RT-qPCR. Data are expressed as mean ± SEM from three independent experiments (*n* = 5 mice per group). ** *p <* 0.01; * *p <* 0.05.

As PD-L2 is involved in the regulation of T cell activation during the infection by *C. hepatica*, we attempted to determine any change in the T cell or macrophage phenotypes related to the aggravated immunopathology after the PD-L2 pathway is blocked in vivo. FCM analyses revealed that CD3^+^IFN-γ^+^ T cells in the spleens of mice infected with *C. hepatica* were significantly increased after being treated with an anti-PD-L2 monoclonal antibody, compared to those without treatment, indicating that the blockade of PD-L2 increases the IFN-γ-expressed Th1 cells in the spleen (Figure 4a). Notably, the IFN-γ transcriptional level was also increased significantly in the liver tissue 6 weeks after treatment with the anti-PD-L2, compared to the control group of mice (i.e., receiving isotype IgG), as measured by RT-qPCR, indicating that the PD-L2 blockade increases the Th-1 response in the livers of infected mice (Figure 4b). Conversely, IL-4 and IL-10 transcriptional levels were reduced in the livers of the mice treated with anti-PD-L2 during the late stage of infection (9–12 weeks post-infection; Figure 4c,d). As expected, the blockade of the PD-L2 with the anti-PD-L2 antibody significantly switched M2 macrophages to M1 in the liver tissue of the infected mice. The infection-induced M2 macrophages expressing Arginase-1 were significantly reduced upon treatment with the PD-L2 antibody (Figure 4f), and infection-inhibited M1 macrophages expressing iNOS were recovered, especially during the late infection period (9–12 weeks post-infection; Figure 4e), in comparison with the infected mice without treatment.

## 4. Discussion

After long-term evolution in mammalian hosts, helminths have developed strategies to survive in the hostile immune environment. It has been shown that helminths have acquired the ability to modulate host immune responses, especially through skewing host immune response to the type-2/regulatory phenotype, in order to escape Th-1-based immune attack [21,22,23]. Macrophages play important roles in both innate immune responses and adaptive immunity, as one of the first lines of defense against pathogens [24]. At the beginning of the infection, classically activated macrophages (or M1) are activated to phagocytize and clear the invading pathogens, creating a pro-inflammatory microenvironment dominated by Th-1 cytokines, such as INF-γ and TNF-α cytokines, which also causes tissue injury as a consequence [25]. In the meanwhile, the macrophages also play a role in tissue repair and wound healing by polarizing to M2 (or alternatively activated macrophages). The M2 macrophages are capable of anti-inflammatory responses dominated by the secretion of IL-10 and TGF-β [26]. Many studies have revealed that helminth infection or helminth-derived products enable the induction of M2 macrophage polarization as a critical pathway or consequence of helminth infection-induced immunomodulation, with beneficial effects to both the parasite and host, including reduced inflammatory response and tissue damage [27,28,29,30]. The differential activation of macrophages during helminth infections determines the pathological outcome of the infection [27]. Late-stage (or chronic) infection with helminths usually induces M2 macrophages, along with functional repair of tissue damaged by the parasites [27]. In this study, we identified, for the first time, that the late-stage infection of *C. hepatica,* specifically regarding the egg-derived products, induced M2 macrophage polarization in the livers of the infected mice, associated with reduced or limited liver damage caused by the egg granulomas.

*Capillaria hepatica* is regarded as a seriously neglected zoonotic parasite, which infects the liver of mammalian hosts and causes fibrosis or even hepatic failure. Most of the published studies on capillariasis have described the epidemic pattern in rodent infections or the clinical features in human patients from sporadic cases [1]. To date, there have been few reports focusing on the immune response or regulation mechanism involved with a *C. hepatica* infection. In this study, we observed, for the first time, that macrophage activation and the polarization from M1 to M2 dynamically occurred during the whole infection course of *C. hepatica* in a mouse model. At the early stage of infection (1–3 weeks post-infection), M1 macrophages were activated in the infected liver, which may be attributed to the protective immunity against infection [25]. However, after the adult worms developed and started to lay eggs in the liver parenchyma in the third week post-infection, M2 macrophages were induced in the liver, which may facilitate parasitism and egg production in the host. The shift from M1 to M2 during the infection course was correlated with the secretion of Th1 cytokines (IFN-γ and TNF-α), changing to Th2 cytokines (IL-10 and IL-13), further indicating that M1 is related to the pro-inflammatory response and M2 to the Th2/regulatory response. Further in vitro study in the RAW246.7 macrophage cell line identified that PD-L1 was stimulated by the adult antigens of *C. hepatica* only on the M1 macrophages, while PD-L2 was induced by the egg antigens on the M2 macrophages, which is correlated with the in vivo infection course of *C. hepatica*. The data were in accordance with previous studies noting that eggs of hepatotropic helminths, such as *S. japanicum* [7], *S. mansoni* [8], and *F. hepatica* [9], were metabolically active and antigenic, which enabled them to induce M2 polarization.

Programmed death 1 (PD-1) and its ligands (PD-L1 and PD-L2) play an important role in regulating T cell activation, tolerance, and immunopathology, during an infection or neoplastic diseases, through the delivery of inhibitory signals [31]. In parasitic infections, PD-1/PD-L1 or PD-1/PD-L2 signaling has been reported to be involved in the immune responses to protozoa, nematode, trematode, and cestode infections [32]; however, the effect of a *C. hepatica* infection on the regulation of the PD-1/PD-L1 or PD-L2 signaling pattern has not been previously reported. In this study, we found that PD-1 was activated in splenic CD4^+^ T-cells upon *C. hepatica* infection and was highly boosted at the late stage of infection (6 weeks post-infection), indicating the *C. hepatica* infection induces immune regulation in the host, especially after the adult worms have developed and eggs have been laid in the infected liver, which is coincident with an increase in M2 macrophages in the infected liver at the same time (Figure 1 and Figure 2). The ligands of PD-1—that is, PD-L1 and PD-L2—were also strongly stimulated on the surfaces of the macrophages in the infected liver; however, they presented a totally different dynamic pattern during the infection. PD-L1 was induced in the early infection and reached its highest level in the 3rd week after infection, correlating with the adult worm development and the increase in M1 macrophages in the infected liver (Figure 2). Meanwhile, PD-L2 was mainly induced in the late stage of infection (after 3 weeks of infection) and reached a peak at the 12^th^ week—the period when eggs had been laid in the liver and the M2 macrophages were induced. The concurrency of PD-L1 and M1, as well as PD-L2 and M2, in the liver macrophages during the course of the infection prompted us to determine any correlation between the macrophage phenotypes and the PD-L1/PD-L2 expression. Interestingly, the PD-L1 was mostly expressed on the surface of the M1 macrophages (CD86^+^) during the early infection (1–3 weeks), but not on the CD206^+^ M2 macrophages, while the PD-L2 was mostly expressed on the CD206^+^ M2 macrophages during the late stage of infection (Figure 2f,g). An in vitro experiment with the RAW246.7 macrophage cell line also revealed that PD-L1 was mainly stimulated by *C. hepatica* adult antigens on CD86^+^ cells (M1), while PD-L2 was highly induced by egg antigens on CD206^+^ cells (M2); see Figure 2h,i. Both in vivo and in vitro experiments confirmed that PD-L1-expressing M1 macrophages were stimulated during the early infection stage before the adult worms had developed in the liver. After the adult worms started to lay eggs in the liver parenchyma, the egg-secreted antigens stimulated PD-L2 expression on the M2 macrophages, which may regulate the formation of egg granulomas and facilitate the survival of eggs or adult worms in the infected liver. Even though PD-L1 was induced on the surface of the M1 macrophages at the early stage of infection, PD-1 expression was not significantly induced on CD4^+^ T-cells at the same time, until the eggs appeared in the liver at 6 weeks after infection. Therefore, we suggest that the PD-1/PD-L2 pathway, rather than PD1/PD-L1, is mostly involved in the immunomodulation induced by a late *C. hepatica* infection. It has been previously observed that PD-L1 and PD-L2 are regulated by M1 and M2 macrophages, respectively [26].

PD-L1 is mostly expressed on T and B cells, macrophages, and dendritic cells, while PD-L2 is typically expressed in DC and on macrophages induced by alternative activation [33,34,35]. Our results were consistent with reports indicating that M2 macrophages were involved in allergic reactions and chronic parasitic infections [15,30]. Many studies have reported that the PD-1/PD-L1 checkpoint pathway is involved in the immunomodulation induced by helminth-stimulated M2 [15,30], but little has been reported regarding the induction of PD-L2 in helminth infections. Here, we identified, for the first time, that a *C. hepatica* infection—especially the egg-derived antigens—induced PD-L2-expressing M2 macrophages, which may be involved in the survival of the parasitic worms or eggs, or the immunopathology in infected liver tissue.

To further investigate the regulatory role of PD-L2 in the *C. hepatica* infection, an anti-PD-L2 monoclonal antibody was used to block the PD1/PD-L2 checkpoints soon after the mice were infected with the *C. hepatica* embryonated eggs. Surprisingly, the blockade of PD-L2 in the infected mice reversed the infection-induced Th2 responses back to Th1 immune responses, with significantly increased IFN-γ-expressing T cells in the splenocytes and IFN-γ transcriptional level in the infected liver tissue, as well as decreased levels of IL-4 and IL-10, in parallel with increased M1-produced iNOS and decreased Arginase-1 expression on the M2. These results clearly demonstrated that the PD-1/PD-L2 pathway is involved in the *C. hepatica* infection-induced M2 macrophage-related immunomodulation, as the blockade of this pathway restored the Th1-biased immune response to the *C. hepatica* infection and shifted the M2 back to the M1 phenotype. However, the results obtained in this study differed from the observation regarding infection with the nematode *Nippostrongylus brasiliensis* in mice, in which a PD-L2 blockade caused an enhanced Th2 response and higher M2 functionality [32]. The blockade of PD-L2 in mice infected with *Trypanosome cruzi* also increased the Arginase I expression in M2 and decreased iNOS expression in M1 macrophages, facilitating the survival of the protozoan in the host [36]. However, in cutaneous leishmaniasis, anti-PD-L1 treatment, but not anti-PD-L2, significantly increased IFN-γ-producing CD4^+^ and CD8^+^ T cells, with significantly lower parasite loads but bigger lesions [34]. These controversial results in different parasite models may reflect the different immune responses or regulatory mechanisms, and the PD-1/PD-L1 or PD-L2 pathways may play different roles in controlling different parasite infections or parasite-caused pathologies.

To determine the effect of the blockage of PD-L2 on the pathology caused by the egg granuloma in the livers of infected mice, the mice infected with *C. hepatica* were treated with an anti-PD-L2 antibody. Surprisingly, the treatment with anti-PD-L2 had no effect on egg reduction in the liver but exacerbated the pathology caused by the egg-formed granulomas in the infected liver, associated with higher inflammatory cell infiltration and deteriorated fibrosis. The possible mechanism behind this finding is that blockade of the PD-1/PDL2 pathway reverses the helminth infection-induced regulatory M2 activation back to M1 activation, which causes the Th1 cellular response to dampen the inflammation, egg granuloma, and fibrosis in the infected liver. The increased Th1 cellular response may have no effect on the eggs within the granuloma, due to the thick wall of collagen surrounding the eggs providing protection.

Taken together, our study demonstrated that a *C. hepatica* infection modulates the host immune response, mostly due to the egg-derived antigens, in order to promote M2 macrophage polarization and PD-L2 expression. The PD-L2-expressing M2 macrophages play important roles in maintaining the Th2-biased and regulatory immune responses, which may facilitate the survival of the parasitic worms or eggs within the infected liver while reducing the pathology caused by the egg granulomas. Treatment with an anti-PD-L2 antibody had no effect on the survival of the parasitic eggs but deteriorated the pathology of the egg granulomas. These results suggest that PD-1/PD-L2 signaling, which is involved in alternative macrophage polarization, determines the immune response pattern and the immunopathology, therefore determining the outcome of the parasitic infection.

## Data Availability

The data presented in this study are available on request from the corresponding author.

## Supplementary Materials

The following supporting information can be downloaded at: https://www.mdpi.com/article/10.3390/tropicalmed8010046/s1, Figure S1: Dynamic expression of PD ligands on macrophages by flow cytometry co-cultured with AWE and EE in vitro.
