# Supplementary material for: PD-L2 Blockade Exacerbates Liver Lesion in Mice Infected with Capillaria hepatica through Reducing Alternatively Activated Macrophages
Source: Trop Med Infect Dis. 2023 Jan 6;8(1):46. doi: 10.3390/tropicalmed8010046 (PMC9866821; doi:10.3390/tropicalmed8010046)
Supplement: Supplementary file 1 [file tropicalmed-08-00046-s001.zip › tropicalmed-2088444-supplementary.pdf]

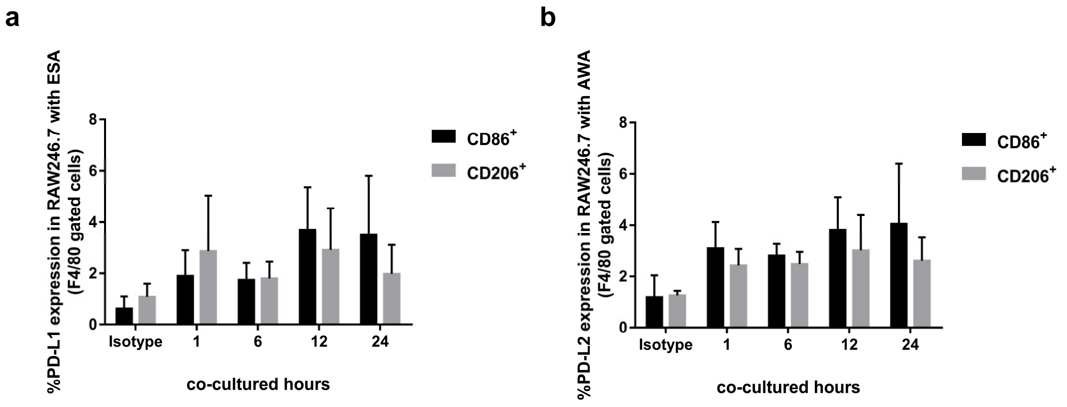

Figure S1. Dynamic expression of PD ligands on macrophages by flow cytometry co-cultured with AWE and EE *in vitro*: (**a**) PD-L1 expression on M1 ($F4/80^+CD86^+$) and M2 ($F4/80^+CD206^+$) RAW246.7 cell line co-incubated with EE *in vitro*. (**b**) PD-L2 expression on M1 ($F4/80^+CD86^+$) and M2 ($F4/80^+CD206^+$) RAW246.7 cell line co-incubated with AWE *in vitro*. No significant changes were observed, and we indicate the specific expression of different antigens stimulating PD ligands *in vitro*.
